# Distorted Acquisition of Dynamic Events Sensed by Frequency-Scanning Fiber-Optic Interrogators and a Mitigation Strategy

**DOI:** 10.3390/s22062403

**Published:** 2022-03-21

**Authors:** Hari Datta Bhatta, Roy Davidi, Arie Yeredor, Moshe Tur

**Affiliations:** School of Electrical Engineering, Tel-Aviv University, Tel-Aviv 6997801, Israel; roydavidi@tauex.tau.ac.il (R.D.); ariey@tauex.tau.ac.il (A.Y.); tur@tauex.tau.ac.il (M.T.)

**Keywords:** fiber-optic sensing, non-uniform sampling, harmonic distortion, post-processing technique, distortion mitigation

## Abstract

Fiber-optic dynamic interrogators, which use periodic frequency scanning, actually sample a time-varying measurand on a *non-uniform* time grid. Commonly, however, the sampled values are reported on a *uniform* time grid, synchronized with the periodic scanning. It is the novel and noteworthy message of this paper that this artificial assignment may give rise to significant distortions in the recovered signal. These distortions increase with both the signal frequency and measurand dynamic range for a given sampling rate and frequency scanning span of the interrogator. They may reach disturbing values in dynamic interrogators, which trade-off scanning speed with scanning span. The paper also calls for manufacturers of such interrogators to report the sampled values along with their instants of acquisition, allowing interpolation algorithms to substantially reduce the distortion. Experimental verification of a simulative analysis includes: (i) a commercial dynamic interrogator of ‘continuous’ FBG fibers that attributes the measurand values to a uniform time grid; as well as (ii) a dynamic Brillouin Optical time Domain (BOTDA) laboratory setup, which provides the sampled measurand values together with the sampling instants. Here, using the available measurand-dependent sampling instants, we demonstrate a significantly cleaner signal recovery using spline interpolation.

## 1. Introduction

Fiber-optic sensors have become indispensable ingredients in many applications, too wide and diverse to enumerate [1,2,3,4,5]. They can directly measure strain, temperature, electric and magnetic fields, as well as rotation, and indirectly many other measurands in both static and dynamic scenarios. The latter include, for example, the measurement of the temporally varying strain fields in structural health monitoring (SHM) of flying airplanes, traffic carrying bridges and other civil structures, under dynamic loading [6]. Aiming at the detection of a damage at its embryonic stage, it is of prime importance to obtain an accurate and undistorted dynamic signature of the relevant strain field.

A leading sensor for these applications is the Fiber Bragg grating (FBG) [7,8], for which the wavelength (or frequency) of peak reflection, $\lambda_{B}\;\left(\mathrm{or}\;\nu_{B}\right)$, is directly related to the local strain and temperature (henceforth, the ‘measurand’). For sensing purposes, FBGs are written along the fiber, either at discrete locations, or continuously inscribed along the fiber during the manufacturing process [9]. For interrogation, a few techniques have been developed to accurately extract the $\lambda_{B}$ of the tested FBG. A common technique, applicable to both the discrete and continuous cases (though of different complexities, [7,8,10]), involves periodic wavelength scanning of a range of wavelengths, which encompasses the values that $\lambda_{B}$ may acquire under the anticipated loading conditions. During each scan and provided that the scan rate is fast enough (with respect to the temporal bandwidth of the measurand), the interrogator correctly measures the instantaneous reflection spectra of the various concatenated FBGs. These peaks which determine the sought-after $\lambda_{B}^{\prime }$ occur, however, at instants dictated by the actual values of the measurand under study, and are generally not time-synchronized with beginning/end of the scan. For example, let the periodic scan cover a wavelength range which includes the $\lambda_{B}^{\prime }s$ of interest, starting at $\lambda_{start}$ and ending at $\lambda_{end}$. Clearly, a low strain value will have its corresponding $\lambda_{B}$ occur near $\lambda_{start}$, i.e., near the beginning of the scan, while a large strain value will have its $\lambda_{B}$ measured only towards the end of the scan. Thus, despite the periodic nature of the scan, the resulting $\lambda_{B}^{\prime }s$ are actually obtained on a *non-uniform* temporal grid. Nevertheless, commonly available frequency-scanning interrogators report their obtained measurand values (the $\lambda_{B}^{\prime }s$ in the case of FBG interrogation) on a *uniform* time grid, either at the beginning of the scan or at its end.

In this paper, we show for the first time to our knowledge, that reporting the obtained measurand values on a uniform time grid, while they were actually obtained on a non-uniform one, results in both harmonic distortion and time-domain errors. These errors grow in significance: (i) as the dynamic range of the measurand (in terms of its induced wavelength/frequency variations) fills the scanning span; and (ii) as the temporal bandwidth of the measurand approaches the Nyquist bound (i.e., half the scanning frequency). This type of harmonic distortion and time-domain errors may be of particular concern for dynamic frequency-scanning fiber sensing techniques, characterized by a tight trade-off between the scanning rate and scanning span. Following an exposition of the problem via simulations in Section 2, a simple post-processing technique appears in Section 3, where knowledge of the instants of sampling (when available) leads to a significant decrease in both the spurious harmonic levels and time-domain errors (since information on the exact time evolution of the scan is generally not made available, the instants of sampling can be estimated from the reported data). Section 4 presents an experimental corroboration of the simulation’s main predictions, utilizing the frequency-scanning technique of Fast Brillouin Optical Time Domain Analysis (F-BOTDA, [11]). Here, a fully controllable laboratory setup measures the strain of an optical fiber under longitudinal and quite pure sinusoidal vibrations (independently measured by a temporally uniform sampling interrogator). Based on a known digitally generated frequency-scanning waveform, the setup augments the reported sampled strain values with the instants at which they were obtained for different values of scan rate and span. Attributing the sampled values to a uniform temporal grid allows us to compare the measured harmonic distortion with the simulation results of Section 2. Moreover, feeding the measured strain values together with their instants of acquisition to the post-processing technique of Section 3, results in a significantly purer signal recovery. Section 5 investigates the performance of a commercial frequency-scanning dynamic interrogator of ‘continuous’ draw-tower FBGs [9,10], which reports the measured strain values only on a temporally uniform grid. This interrogator trades-off scan rate and scan range: the faster the scan, the narrower the span. As expected, the experimental results show that for a fast scan rate and its associated constrained span, the harmonic distortion worsens with increasing signal frequency and/or strain amplitude. Note that when the instants of sampling are not reported, they cannot be deduced from the measured values without a full knowledge of the scan parameters, which are most often not available. Section 6 discusses the findings and provides valuable conclusions.

## 2. Simulations: Strain Interrogation with Periodic Frequency Scanning

**Figure 1:**
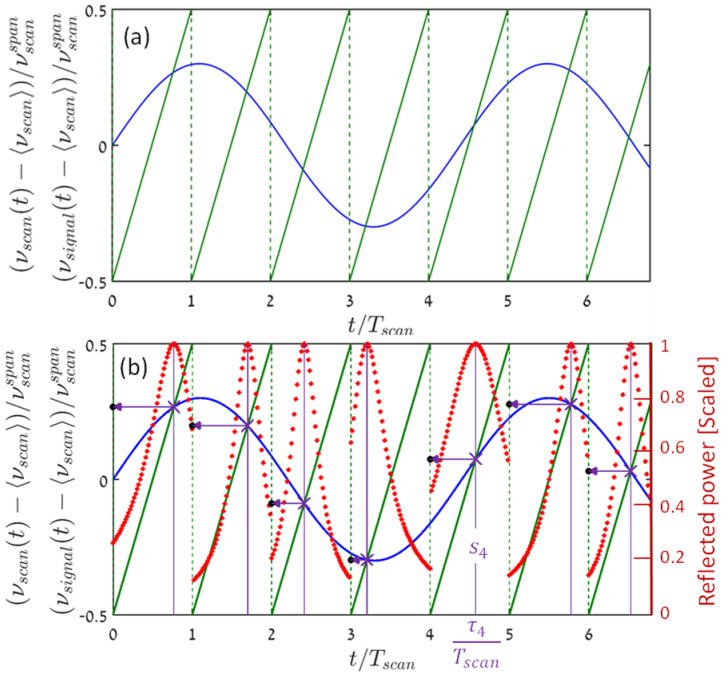
(**a**) A sinusoidal measurand signal of a normalized temporal frequency of $f_{signal}/f_{scan}$ = 0.23 (50 Hz/220 Hz) and filling factor of $\nu_{signal-amp}/\nu_{scan}^{span}=0.3$ (solid-blue sinusoidal curve), is scanned by a periodic (every $\;T_{scan}$ ) saw-tooth waveform (green). (**b**) The saw-tooth scanning results in a time-dependent detected power (FBG reflection [7,8], or Brillouin probe amplification [11,12,13]: red dot curves (arbitrary units). The purple X’s designate the intersection of the signal with the saw-tooth waveform, also indicating the instants where the detected power reaches it maximum. The black filled circles are the measurand sampled values (the ordinates of the X’s), attributed to the beginning of the corresponding scan periods. Only the middle ~6 scan periods are shown from a simulated temporal range of $\left[-550\;549\right]T_{scan}$.

The reflection from an FBG [7,8], as well as the probe gain in Brillouin-based sensing [11,12,13], are frequency dependent, having a characteristic spectral shape, commonly denoted here by S(ν). In both cases, and for a given time t, S(ν) peaks at a frequency, which is a unique function of the value of the measurand at that instant. Let $\;\nu_{signal}\left(t\right)$, of sinusoidal shape, amplitude $\;\nu_{signal-amp}$, and frequency $f_{signal}$, Figure 1a (solid blue), represent that peak frequency, which is the local Brillouin frequency shift (BFS) in Brillouin-based sensing (see Section 4), and $c/\lambda_{B}$ for an FBG (as in Section 5).

The commonly used saw-tooth-type frequency-scanning signal, $\nu_{scan}\left(t\right)$ in Figure 1a (solid green curve), is assumed to be ideal, having a perfect linear ramp and abrupt return, a scan rate of $f_{scan}$ ($=1/T_{scan}$) and peak-to-peak scanning span of $\nu_{scan}^{span}$ (for optimum coverage, the signal mean value is placed at the center of the frequency scan, $\langle \nu_{scan}\rangle$). Both the normalized frequency, $\xi =f_{signal}/f_{scan}$, and filling factor, $\eta =\nu_{signal-amp}/\nu_{scan}^{span}$, can range from near zero (for dense sampling and very small signal amplitude) to below 0.5 (the Nyquist limit, and a meaurand peak-to-peak dynamic range that fills the scan span). The simulation assumes that the signal mean value perfectly aligns with that of the scan, Figure 1. This is optimal for best utilization of the scan span. Anyway, the simulation results are independent of the signal mean, as long as the full dynamic range of the signal lies within that scan span.

As the optical frequency of the interrogator’s source is periodically scanned, either continuously or in small frequency steps, a photodetector measures the frequency-dependent reflected power from the FBG [7,8], or the frequency-dependent power of the Brillouin-amplified probe [11,12,13]. In the simulation, S(ν) is assumed to have a Lorentzian shape, $1/\left[{\left(\nu -\nu_{peak}\right)}^{2}+{\left(∆\nu_{3dB}\right)}^{2}\right]$, characterized by maximum value at $\nu_{peak}$, and a (scaled) full width half maximum of $∆\nu_{3dB}/\nu_{scan}=0.6$. The time-dependent detector power, P(t). can then be expressed by:

(1) $$P\left(t\right)\propto \frac{1}{{\left[\nu_{signal}\left(t\right)-\nu_{scan}\left(t\right)\right]}^{2}+{\left(∆\nu_{3dB}\right)}^{2}}$$

The simulated detector power in the neighborhood of its local peak at each of the scan periods is represented by the red-dot curves in Figure 1b (in arbitrary units). The vertical purple lines in Figure 1b cut the time axis at those instants, ${\left\{\tau_{n}\right\}}_{n=0}^{N-1}$, at which the detector power vs. time records attain their maxima at each scan cycle (see the n=4, fifth period in Figure 1b), where N is the total number of scan cycles. Ideally, in the absence of noise and other perturbations, these maxima occur at the intersections of the signal and the saw-tooth scanning curves (purple X’s in Figure 1b). Mathematically, ${\left\{\tau_{n}\right\}}_{n=0}^{N-1}$ are the solutions of:

(2) $$\nu_{signal}\left(\tau_{n}\right)=\nu_{scan}\left(\tau_{n}\right),\;\;nT_{scan}\leq \tau_{n}<\left(n+1\right)T_{scan},\;\;n=0,\ldots ,N-1$$

Once the intersection points are found, their ordinates are the desired measurand values (see the 5th period):

(3) $$s_{n}\equiv \nu_{signal}\left(\tau_{n}\right)=\nu_{scan}\left(\tau_{n}\right),\;\;n=0,\ldots ,N-1$$

Note that the actual shape of the detector record is not fixed but rather depends on the signal dynamics, and specifically on the slopes of the signal and scan waveform near the instant of intersection. The more parallel the signal and waveform are, the wider the recorded shape and vice versa (cf. the red-dot curves in the second and fifth periods).

In the simulated scenario of Figure 1, sampling rate is more than four times the signal frequency, seemingly more than sufficient for accurate recovery of the signal from its samples.

**Figure 2:**
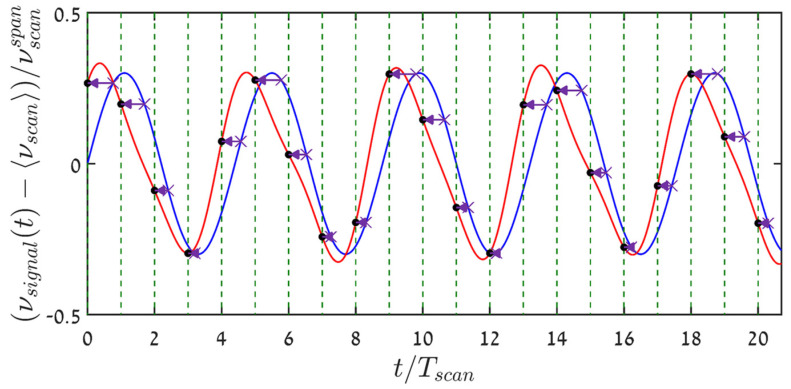
The original sinusoidal signal (blue) of Figure 1, together with its Nyquist–Shannon sinc-reconstruction (red) from the sampled values, ${\left\{s_{n}\right\}}_{n=0}^{N-1}$, assuming the latter are attributed (see horizontal arrows) to the scans’ starting points (vertical dashed green lines). Shown are the middle ~20 scan periods out of 1100, starting at $-550\;T_{scan}$.

However, since the signal varies in time from scan period to another, it is clear from the ramp-type nature of the scanning, as well as from the nonlinear characteristics of Equation (2) and graphically, from Figure 1b, that the sampling instants have variable signal-dependent distances from the beginning of the scan cycles that encompass them. For example: while in the first period of Figure 1 sampling occurs towards the end of the scan, it is just the opposite at the fourth period. Yet, as already mentioned in the introduction, it appears, that frequency-scanning interrogators report their acquired measurand values at uniformly spaced instants, associated with either the beginning, ${\left\{s_{n},nT_{scan}\;\right\}}_{n=0}^{N-1}$ or end of the relevant scan period, ${\left\{s_{n},\;(n+1)T_{scan}\right\}}_{n=0}^{N-1}$. This is bound to result in erroneous reconstructions of the sampled signal. Indeed, high-granularity sinc-reconstruction based on the sampled measurand values, ${\left\{s_{n}\right\}}_{n=0}^{N-1}$ (the ordinate values of the purple X’s)), when attributed to the beginnings of the scans (black full circles in Figure 1b), produces the red curve of Figure 2, which is not only (tolerably) time-shifted, but also a distorted reconstruction of the true signal (blue curve). Note that sinc-based reconstruction of the sine wave from its true signal values at the scans’ starting points, ${\left\{nT_{scan}\right\}}_{n=0}^{N-1}$, rather than from the quite different ${\left\{s_{n}\right\}}_{n=0}^{N-1}$, results in a curve indistinguishable from the blue curve of the figure.

**Figure 3:**
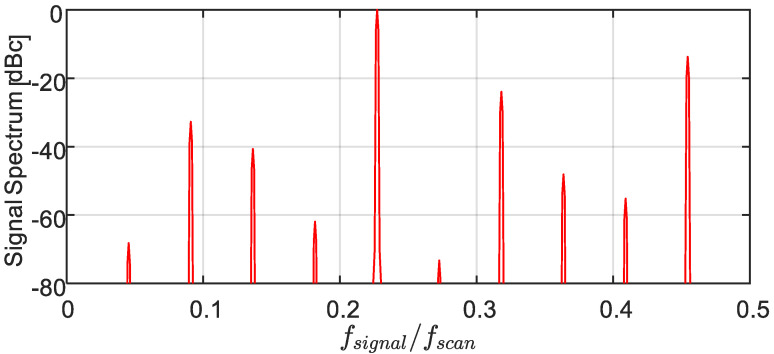
Hamming-weighed FFT-based spectrum of the signal acquired from its per-period intersections with the saw-tooth scanning waveform (Figure 1), exhibiting significant harmonic distortion. The highest harmonic at ξ=0.46 is the signal’s second harmonic, whereas the other peaks are folded ones (see text). The time record was 1100⋅$T_{scan}$ long, starting at −550$\;T_{scan}$. Incidentally, using the true signal values on the same temporal grid of period $T_{scan},$ gives rise only to a single peak at ξ = 0.23.

Assigning the sampled measurand values to a uniform temporal grid makes it possible to spectrally analyze them using DFT/FFT (which implicitly attributes these values to a uniform time-grid). Figure 3 shows the power spectrum of the signal of Figure 1, based on its obtained samples values, ${\left\{s_{n}\right\}}_{n=0}^{N-1}$. Here, the sampling rate is more than twice the Nyquist rate, ξ=0.23<0.5, and the signal variations are well within the scan limits, η=0.3<0.5. Yet, instead of a single peak at the signal frequency, ξ=0.23, the spectrum shows many harmonics, where the one at ξ=0.46 is a very strong second harmonic, only −13.73 dB below the signal. All other peaks are folded harmonics (e.g., the peak at ξ=0.318 is the third harmonic at −23.96 dBc, while the one at ξ=0.136 is the fifth harmonic at −40.69 dBc, etc.). The total harmonic distortion lies at −13.27 dBc. Clearly, the second harmonic is the dominant one. Power leakage to all these harmonics also lowers the peak at the signal frequency by 0.27 dB.

Our simulation assumed an ideal saw-tooth waveform, as well as highly precise determination of the measurand sampled values ${\left\{s_{n}\right\}}_{n=0}^{N-1}$ and their instants of acquisition, ${\left\{\tau_{n}\right\}}_{n=0}^{N-1}$. In practice, however, these values are only estimated from the reflection/gain measurements (red-dot curves in Figure 1) with accuracy affected by (i) the estimation algorithm; (ii) the scanning granularity (the frequency steps); (iii) scan calibration; and (iv) noise. Simulations that include these sources of inaccuracies still exhibit the same strong harmonics in the calculated spectra, quite similar to those of Figure 3. The only exception is the presence of an elevated floor, which masks some of the weaker harmonics (Note that our analysis does not consider the effect of fiber length, since the round-trip time of light in the fiber is assumed to be negligibly small with respect to the scan period).

**Figure 4:**
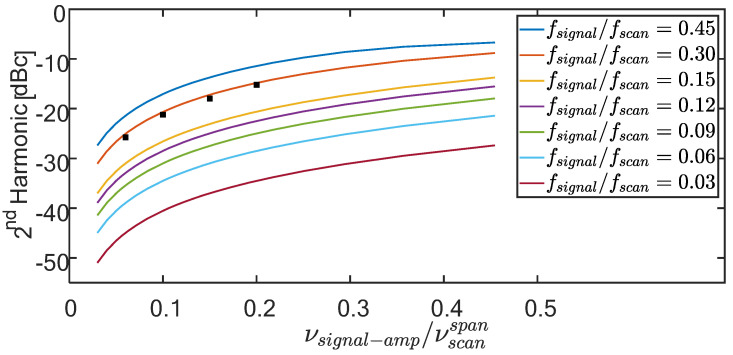
The power (magnitude-squared value) of the second harmonic of an originally pure sinusoidal signal, when acquired from its temporally non-uniform per-period intersections with the linear (instantaneous fly back) saw-tooth scanning waveform of Figure 1. Simulated results are shown for a range of scaled signal frequencies (ξ-legends box) and filling factors (η -abscissa). The higher ξ and/or η, the worse the harmonic distortion. The corresponding total harmonic distortion curves lie within half a dB from the displayed second harmonic ones. The black squares represent experimental results for the Brillouin setup of Section 4. Note that a different scan pattern, such as a triangular one, will result in different curves.

Figure 4 displays simulation results for the dependence of the second harmonic (scaled by the signal power) on$\;f_{signal}/f_{scan}$, and $\nu_{signal-amp}/\nu_{scan}^{span}$ for a sinusoidally varying measurand. Clearly, the larger the measurand amplitude the higher the required scanning frequency for a prescribed amount of harmonic distortion. Conversely, the closer the scanning frequency to the Nyquist rate, the smaller the allowed dynamic range to maintain a maximum permissible level of the second harmonic. Finally, harmonic distortion is a sign of nonlinear behavior of the interrogation process, indicating, for example, that the output of the sum of two inputs will not equal the sum of the individual outputs. Indeed, when the sum of two signals is sampled, the actual sampling instances of the sum are generally different from the actual sampling instances of each of these signals alone; therefore, the sampled values of the sum are generally different from the sum of sampled values of each of the signals. Attributing all samples to the same time grid therefore gives rise to an apparent non-linearity of the sampling mechanism, whereas if all samples are attributed to their correct time instances, linearity is implicitly maintained.

## 3. Distortion Mitigation Based on the Availability of the Sampling Instants

Let us assume that the interrogator reports the sampled values of the measurand, ${\left\{s_{n}=s(\tau_{n})\right\}}_{n=0}^{N-1}$, together their non-uniformly spaced instants of acquisition, ${\left\{\tau_{n}\right\}}_{n=0}^{N-1}$, Equations (2) and (3). While accurate signal recovery from non-uniformly time-spaced samples is not generally possible [14,15], yet, a sharp increase in the fidelity of the reconstructed signal can be achieved using interpolation.

Looking back at Figure 1a, we note that since the mean value of our example signal coincides with the mean of the scan, the sampling instants, ${\left\{\tau_{n}\right\}}_{n=0}^{N-1}$, also gather around the (temporal) middle of the scan: ${\left\{\left(n+0.5\right)T_{scan})\right\}}_{n=0}^{N-1}$. More generally, let us define a shifted uniform temporal grid by:

(4) $${\hat{\tau }}_{n}=nT_{scan}+\underset{n}{\mathrm{mean}}\left(\tau_{n}-nT_{scan}\right),n=0,\ldots ,N-1$$

**Figure 5:**
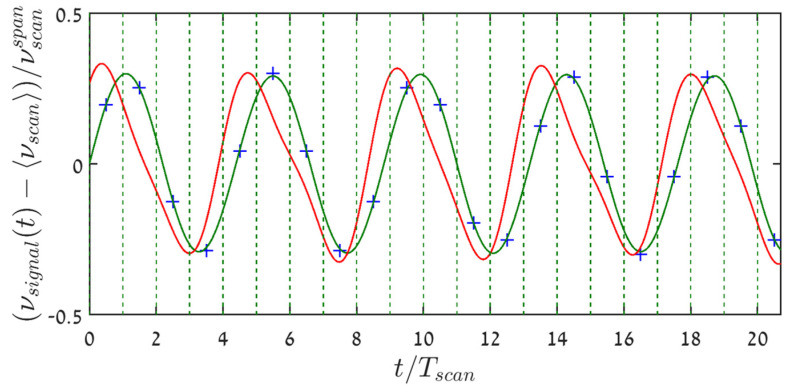
The sinusoidal green curve represents sinc-based reconstruction of the signal from ${\left\{{\hat{s}}_{n},{\hat{\tau }}_{n}\right\}}_{n=-550}^{549}$, where ${\left\{{\hat{s}}_{n}\right\}}_{n=-550}^{549}$ are the spline-interpolated signal values on the computable uniform time grid ${\left\{{\hat{\tau }}_{n}\right\}}_{n=-550}^{549}$, Equation (4). The blue pluses (+) represent a few values of the original sinusoidal signal, and their very tight proximity to the recovered green curve attests to the high quality of the reconstruction. The red curve is the one from Figure 2, representing sinc-based reconstruction from ${\left\{s_{n}\right\}}_{n=0}^{N-1}$, being attributed to the uniform time grid at the start of the scans.

**Figure 6:**
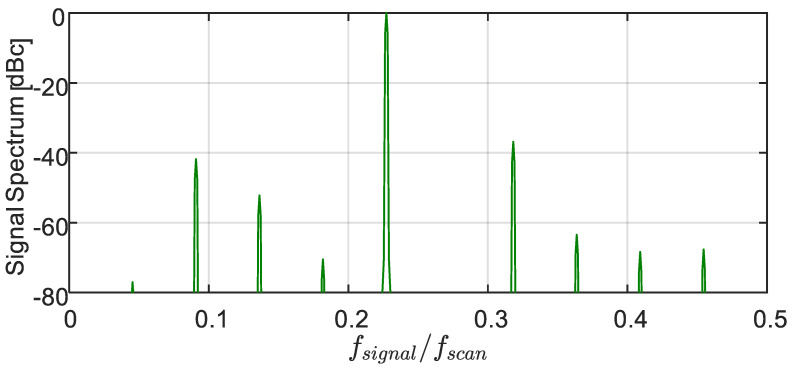
Spectrum of the pure sinusoidal signal of Figure 1, calculated from ${\left\{{\hat{s}}_{n},{\hat{\tau }}_{n}\right\}}_{n=-550}^{549}$, where $\left\{{\hat{s}}_{n}\right\}$ are spline interpolated signal values on the computable uniform time grid ${\left\{{\hat{\tau }}_{n}\right\}}_{n=-550}^{549}$, Equation (4), based on the known sampled values $\left\{s_{n}\right\}$ and their actual sampling instants ${\left\{\tau_{n}\right\}}_{n=-550}^{549}$. Note the considerably lower harmonics, when compared with those of Figure 3.

Interpolation algorithms can now be applied to the measured data pairs, ${\left\{s_{n},\;\tau_{n}\right\}}_{n=0}^{N-1}$, in order to estimate the signal values on the uniform grid of Equation (4): ${\left\{{\hat{s}}_{n}=s\left({\hat{\tau }}_{n}\right)\right\}}_{n=0}^{N-1}$. Thus, spectral analysis and time-domain recovery of the measured signals will now be based on ${\left\{{\hat{s}}_{n},{\hat{\tau }}_{n}\right\}}_{n=0}^{N-1},\;$rather than on ${\left\{s_{n},\;nT_{scan}\right\}}_{n=0}^{N-1}$. The results of processing the full data behind Figure 1 in this manner, using Spline interpolation, are shown in Figure 5 (time domain, where, again, sinc-based reconstruction was used to obtain the much denser displayed granularity), and in Figure 6 in the frequency domain. The benefits of having access to the sampling instants are obvious: the folded third harmonic (ξ=0.318) is now the dominant one but at a level 37 dB below that of the signal. As for the time traces, the standard deviation of the difference between the true signal values at ${\left\{{\hat{\tau }}_{n}\right\}}_{n=0}^{N-1}\;$of Equation (4) and their spline interpolated values, ${\left\{{\hat{s}}_{n}\right\}}_{n=0}^{N-1}$, normalized by the standard deviation of the signal, is a mere 0.02.

## 4. Frequency Scanning Interrogation with Brillouin Fiber-Optic Sensing

### 4.1. Brillouin Optical Time Domain Analysis (BOTDA)

**Figure 7:**
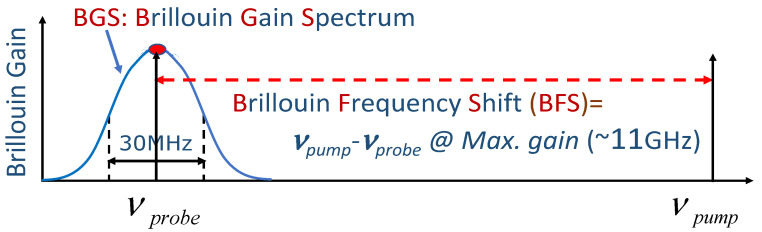
Brillouin amplification in single-mode fibers. Pump light at an arbitrary frequency/wavelength, e.g., $\lambda_{B}$ = 1550 nm, propagating in one direction in the fiber core generates narrowband gain for light propagating in the opposite direction. Gain is maximized when the frequency difference, $\upsilon_{pump}-\upsilon_{probe}$, equals the so-called Brillouin Frequency Shift, $\upsilon_{BFS}$. For silica-based single-mode fibers around 1550 nm, $\upsilon_{BFS}$ is ~11 GHz and the Brillouin gain bandwidth is ~30 MHz for pulses longer than ~40 ns. Of crucial importance for sensing applications is the fact that $\upsilon_{BFS}$ is a function of both strain and temperature, mainly through the dependence of the local acoustic velocity, $V_{A},$ on these two measurands.

In BOTDA [11,12,13], a pump pulse of optical frequency $\upsilon_{pump}$ propagates against a continuous wave (CW) probe of frequency $\upsilon_{probe}$ in a standard single-mode optical fiber. Mediated by an acoustic field, induced by these two waves via interference and electrostriction, the pump pulse can amplify the counter-propagating probe through the coherent process of stimulated Brillouin amplification. Brillouin gain experienced by the probe, Figure 7, exists only in a narrowband of frequencies centered at an optical frequency, downshifted from the pump frequency by a characteristic value, $\upsilon_{BFS}$, called the Brillouin Frequency Shift (BFS). Its value is around 11 GHz for standard single-mode fibers near 1550 nm. It is the linear dependence of $\upsilon_{BFS}$ on the local strain (50 MHz/1000 micro strains) and temperature (1 MHz/°C), that makes Brillouin sensing such a useful technique.

Similar to radar, localization is achieved by correlating the power of the emerging probe at a given time with the moment of launch of the pump pulse. In standard BOTDA setups the spatial resolution is of the order of half the pump pulse length. The BFS is found by measuring the dependence of the local Brillouin gain on the scanning frequency difference, $\upsilon_{pump}-\upsilon_{probe}$, through periodic scanning of the latter, to produce the local Brillouin Gain Spectrum (BGS) of Figure 7. Once the BGS is obtained, the frequency location of its peak determines the BFS, from which the value of the relevant measurand is deduced. For long distance Brillouin interrogation (~kilometers) many pulses are sent out for the same pump-probe frequency difference to facilitate averaging for noise reduction. This makes long-range BOTDA unsuitable for dynamic sensing. However, for short distances (100’s of meters) fast Brillouin-based techniques (e.g., F-BOTDA [12], BOCDA [16]) are the only currently available fiber-optic distributed sensing techniques that can achieve many kHz of sampling rates (limited mainly by time of flight of light in the fiber) with spatial resolution of the order of 10 cm [12,13] over short lengths (tens of meters) of fiber. Brillouin sensing is an active research field with many variants other than BOTDA. For recent reviews see [12,13].

### 4.2. Experimental Setup

**Figure 8:**
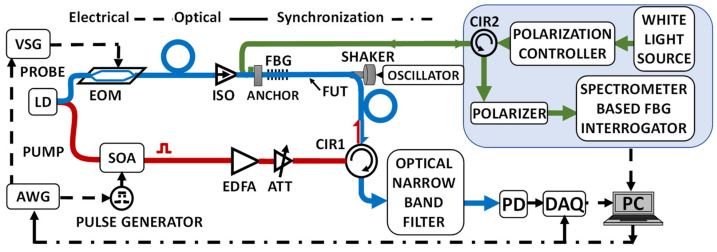
A longitudinally vibrating fiber is interrogated by either an all-polarization-maintaining F-BOTDA setup, producing temporally non-uniform samples, or by a uniformly sampling white-light spectrometer-based interrogator that measures the response of an on-fiber FBG. The $\lambda_{B}$ of the inscribed FBG is away from the Brillouin scanning region. AWG: Arbitrary Waveform Generator, RF: Radio frequency, SOA: Semiconductor Optical Amplifier/switch, EDFA: Erbium Doped Fiber Amplifier, ISO: Optical isolator, FBG: Fiber Bragg Grating inscribed on the FUT, PD: Photo diode, CIR: Circulator, EOM: Electro-Optic Modulator, LD: Narrowband Laser Diode, DAQ: Data Acquisition, VSG: Vector Signal Generator, ATT: Attenuator.

The experimental setup for the Brillouin experiment is shown in Figure 8. The Fiber Under Test (FUT) comprises a 3 m of polarization maintaining (PM) fiber, out of a of ~20 m of non-vibrating PM fiber leads. The FUT is longitudinally vibrated at 50 Hz by a sinusoidally driven shaker, against an anchor point on its other end.

**Figure 9:**
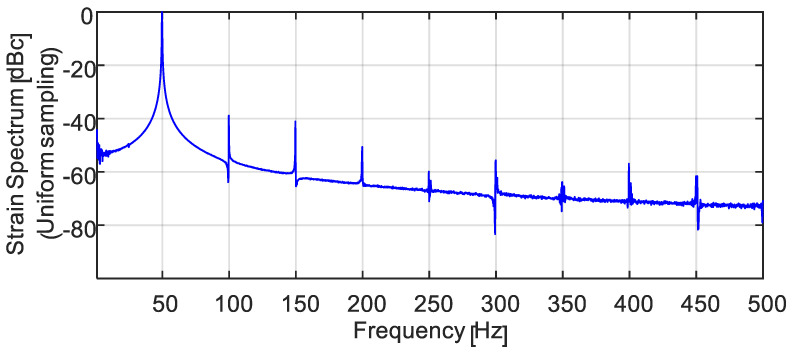
Strain signal spectrum at 50 Hz longitudinal vibrations, interrogated by the spectrometer-based temporally uniform interrogator. Highest harmonic (at 100 Hz) is >37 dB below the signal.

To ensure the vibrations are of high harmonic purity, an FBG, reflecting at 1528 nm, is imprinted on the FUT end, near the fixed anchor, using a frequency-doubled Argon laser and a phase mask. Being a PM fiber, the resulting FBG has slightly different reflection peaks, $\lambda_{B}^{\prime }s$, for the slow and fast axes of the fiber. A commercial, spectrometer-based interrogator was used (shaded box in Figure 8), where the vibration-modulated reflected light from the on-FUT FBG is spectrally analyzed (wavelength-wise) by a diffraction grating onto a fast diode array. Uniformly triggered at a rate of 3 kHz, the diode array collected light for a very short snapshot of 50 μs per trigger (The polarization controller following the white-light source and the polarizer preceding the spectrometer are used to optimally get reflection only from the slow-axis grating of the PM fiber). The FBG interrogator then reports its acquired, time-dependent $\lambda_{B}^{\prime }s$ on a uniform temporal grid in units of strain. FFT-based spectral analysis of the FBG-measured vibrations, Figure 9, confirms that for vibration amplitudes of up to the tested value of 330 microstrains, the longitudinal vibrations of the FUT exhibit very low harmonics (highest one at 100 Hz is more than 37 dB below the 50 Hz peak).

The frequency-scanning Brillouin interrogation is based on an F-BOTDA setup [11], modified in the current setup so that all connecting fibers and components are polarization-maintaining to avoid the need to deal with polarization fading. Here, the output of a coherent <15 kHz in linewidth) CW laser at 1550 nm is split into two arms. Light in the bottom one (red) is carved by a high-extinction-ratio semiconductor optical amplifier (SOA) to generate a 15 ns pump pulse (providing a spatial resolution of 1.5 m), which is then amplified before feeding the FUT through the circulator. The top (blue) arm prepares the probe wave, whose frequency is downshifted from that of the pump by a LiNbO_3_ EOM modulator, driven by a microwave generator (VSG), having I/Q inputs. To achieve fast scanning with almost instantaneous transition between scanning frequencies, the I/Q inputs of the VSG are fed by two-channels of an Arbitrary Waveform Generator (AWG). Frequency scanning around ~10.63 GHz (the static BFS of the slow-axis of the slightly pre-tensioned FUT) is performed by [17]: (i) Setting the VSG center frequency a few hundred MHz above the fiber static BFS; and (ii) feeding the I/Q inputs of the VSG with a Hilbert pair of signals (sine and cosine), comprising a concatenation of sinusoidal segments, each of a fixed frequency, stepping in value from segment to segment by the scan granularity of Δf (2 MHz in our experiments). The length of each segment should be equal or longer than twice the length of the fiber (from the isolator to the circulator, CIR1). The number of such steps times Δf equals the scan dynamic range, ${\bar{\nu }}_{scan}$, which must encompass the frequency range subtended by vibrating BGSs of the FUT. The result of this modulation scheme is a time-efficient periodic, stepwise saw-tooth frequency scanning of a pre-chosen rate, $f_{scan}$ [Hz], inherently limited by the fiber length and scanning granularity.

Following double sideband, suppressed carrier modulation, the probe wave enters the FUT, where it is Brillouin amplified with a gain of 1.1–1.5 dB. Emerging from the FUT through a circulator, CR1, the probe wave is optically filtered to remove the upper sideband, as well as other unwanted signals. The probe power is then detected by a wideband photodiode, whose output is digitized by a deep memory, 1 GSamples/s DAQ, capable of storing seconds-long events. For each pump pulse, the DAQ records the Brillouin gain along the FUT for one value of the pump-probe frequency difference, sampling the length of the FUT every 1 ns (i.e., every 10 cm). One complete scan-cycle comprises enough pump pulses and different probe frequencies, $\left\{\nu_{k},\;k=1,\ldots K\right\}$, to cover the scan span. Thus, the recorded data for the n-th scan-cycle, contains all necessary information to calculate the Brillouin gain, $Gain\left(t=n/f_{scan},{\left\{\nu_{k}\right\}}_{1}^{K},z_{m}\right)$, for each scanning frequency, $\nu_{k}$, and fiber locations, $\left\{z_{m}\right\}$, spaced by 10 cm for a DAQ sampling frequency of 1 ns (spatial resolution is still 1.5 m for a pump pulse width of 15 ns). The time-varying Brillouin frequency shift, $BFS\left(t=n/f_{scan},z_{m}\right)$, is then estimated from $Gain\left(t=n/f_{scan},{\left\{\nu_{k}\right\}}_{1}^{K},z_{m}\right)$ by: (i) fitting a parabola to the top 30% values of the dependence of $Gain\left(t=n/f_{scan},{\left\{\nu_{k}\right\}}_{1}^{K},z_{m}\right)$ on ${\left\{\nu_{k}\right\}}_{1}^{K}$ [18]; (ii) estimating the instants, {$\tau_{n}\left(z_{m}\right)\},$ along the n-th scan when the peak of the parabola (i.e., maximum gain) was reached; and (iii) use the known AWG-governed, precise slope of the saw-tooth scanning, to calculate $BFS\left(t=n/f_{scan},z_{m}\right)$, which is the pump-probe frequency difference at $\tau_{n}\left(z_{m}\right)$. Converting $BFS\left(t=n/f_{scan},z_{m}\right)$ to strain values and collecting the results of many sequential scans, the value of the measurand can be estimated as a function of time for all points along the FUT.

### 4.3. Brillouin Sensing Results

Before proceeding to the obtained results (preliminary version of them appeared in [19]), we note that measurements under static conditions produced BFS values with a standard deviation of less than 1 MHz. In addition, the vibration amplitude was measured to be constant along the FUT (within the measurement accuracy), indicating a single-longitudinal-mode behavior.

**Figure 10:**
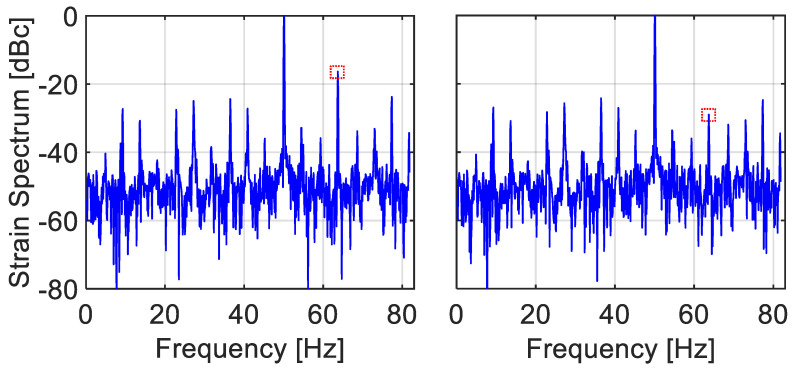
(**Left**) Hamming-weighed FFT spectrum of raw BFS vs. time 50 Hz vibration data, obtained from the experimental setup of Figure 8. The scan rate is 164 Hz, the scan range is 108 MHz and the vibration amplitude is 17 MHz, resulting in $f_{signal}/f_{scan}$ = 0.3 and $\nu_{signal-amp}/\nu_{scan}^{span}$ = 0.16, respectively. Note the strong (−16.3 dB) second harmonic (dotted square), occurring at the folded frequency of 64 Hz (=164/2 − (2 × 50 − 164/2)). It is due to the fact the FFT algorithm implicitly treats its input temporally non-uniform data as uniform (The other peaks are folded higher harmonics). (**Right**) Using the measured instants of acquisition, ${\left\{\tau_{n}\right\}}_{n=0}^{N-1}$, and the procedure of spline interpolation of Section 3, the second harmonic is significantly attenuated to −29 dB (dotted square). Record duration is 9 s.

**Figure 11:**
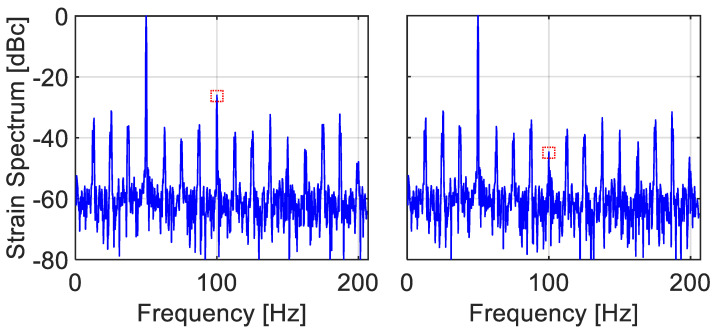
(**Left**) Hamming-weighed FFT spectrum of raw BFS vs. time 50 Hz vibration data, obtained from the experimental setup of Figure 8. Here, the scan rate is 412 Hz, the scan range is 112 MHz and the vibration amplitude 17 MHz, resulting $f_{signal}/f_{scan}$ = 0.12 and $\nu_{signal-amp}/\nu_{scan}^{span}$ = 0.15, respectively. Note the −26 dB second harmonic peak at 100 Hz (dotted square). While lower than the −16.3 dB one of Figure 10, it is still higher than the spectrometer-based measurement of below −37 dB (The observed peaks are again folded high harmonics). (**Right**) Using the measured instants of acquisition, ${\left\{\tau_{n}\right\}}_{n=0}^{N-1}$, and the procedure of spline interpolation of Section 3, the second harmonic is down to −44.7 dB (dotted square) but there is now a dominant harmonic at −31 dB. Record duration is 3.6 s.

Figure 10 and Figure 11 present FFT-based spectral analysis of the experimentally obtained strain signals under 50 Hz of longitudinal vibrations, using BFS vs. time data, $\left\{s_{n}\right\}$ from the middle of the FUT. Two different acquisition conditions are presented in the figures: the case of relatively large values of $\xi =f_{signal}/f_{scan}=$ 0.3 and $\eta =\nu_{signal-amp}/\nu_{scan}^{span}$ = 0.16 appear in Figure 10, while the case of lower values of ξ= 0.12 and η= 0.15 is shown in Figure 11. Since our experimental setup is based on frequency scanning, the obtained BFS values are actually measured on a non-uniform temporal grid, ${\left\{\tau_{n}\right\}}_{n=0}^{N-1}$. FFT spectral analysis, however, implicitly assumes the data sit on a uniformly spaced one.

Therefore, two approaches are tried:

1. The easiest and, apparently, also the commonly followed approach: Artificially assign the measured data to a uniform temporal grid, synchronized with the periodic scanning, e.g., the starting or ending points of the scan. The result of treating the data as if they represent uniform sampling appears on the left panels of Figure 10 and Figure 11. The exhibited second harmonic levels are much higher than those present in the actual vibrations, as verified by the much faster and uniformly triggered FBG interrogator, Figure 9. Note that the observed second harmonic levels in these two scenarios (−16.3 and −26 dB, respectively), as well as in a few other scenarios, are in good agreement with the simulation results of Figure 4 (superimposed black squares).
2. Take advantage of the availability in our experiment of the instants of sampling, ${\left\{\tau_{n}\right\}}_{n=0}^{N-1}$, and use interpolation to estimate the values of the measurand on a uniform time grid. Employing the spline-based procedure of Section 3, the right panels of Figure 10 and Figure 11 show substantial attenuation of the second harmonic to −29 and −44.7 dB, respectively (although other harmonics now become the dominant ones in the right panels (at −24.8 dB in Figure 10 and −31.6 dB in Figure 11)).

Thus, the flexible laboratory setup of this section has allowed us to obtain access to the sampling instants, thereby corroborating two aspects of the simulation results of Section 2 and Section 3: (i) assigning the temporally non-uniformly spaced measured values to a uniform time grid results in errors; and (ii) these errors can be significantly mitigated using interpolation techniques, but only if the sampling instants are made available.

## 5. Frequency Scanning Interrogation of Draw-Tower FBGs

### 5.1. ‘Continuous’ FBG Fibers and Their Interrogation

Modern technology enables the writing of Bragg gratings while the fiber is being drawn [9]. Our experiment uses draw-tower ‘continuous’ FBG (CFBG) fiber of this type, which comprises a periodic repetition of a 9 mm-long, very weak FBGs (reflectivity~0.0001), with a gap of 1 mm. Since all gratings are nominally inscribed to have the same wavelength of peak reflection, Optical Frequency Domain Reflectometry (OFDR) [10,20] is commonly used to recover the $\lambda_{B}$’s (i.e., the wavelengths of peak reflection) of different spatial resolution cells along the fiber. In this technique, a highly coherent tunable laser frequency periodically scans the CFBG fiber with light of complex amplitude, $A_{in}\left(\nu \right)$, and the reflected light, $A_{reflected}\left(\nu \right)$, is made to interfere with a reference derived from the same laser. The resulting measured power involves an interference term that contains all necessary information needed in order to calculate the complex-valued (amplitude and phase) Optical Transfer Function (OTF), $T\left(\nu \right)=A_{reflected}\left(\nu \right)/A_{in}\left(\nu \right)$, of the whole CFBG fiber over the scanning span. Once measured, T(ν) can be Fourier transformed into the time domain (where time is related to distance into the fiber, z) to obtain the impulse response of the fiber, IR(z), representing the (complex-valued) return from the vicinity of z. Cutting a segment from IR(z), say around $z_{0},$ and inverse-Fourier transforming it back into the frequency domain, gives us the reflection spectrum of the FBG at $z_{0}$, from which the local wavelength of peak reflection, $\lambda_{B}\left(z\right)$, can be estimated. This illustrative procedure, or rather its proprietary commercial implementations, using modern powerful DSP processors, are then used for the calculation of the measurand-induced shift for each spatial resolution cell along the fiber (spatial resolution is governed by the width of the frequency span).

### 5.2. The FUT Tested by the Commercial Interrogator

The experiment reported below used a commercial dynamic and high spatial resolution (<1 cm) distributed strain/temperature interrogator, which trades-off scan rate with scan range. While at low scan rates the instrument has an extremely wide scanning span, at its highest scan rate the span is significantly narrower. Being a frequency-scanning interrogator, the instrument actually captures the values of a dynamic measurand on non-uniformly spaced instants, yet it reports the sampled values on a temporally uniform grid, with no access to information about when, during the scan, those values occurred.

**Figure 12:**
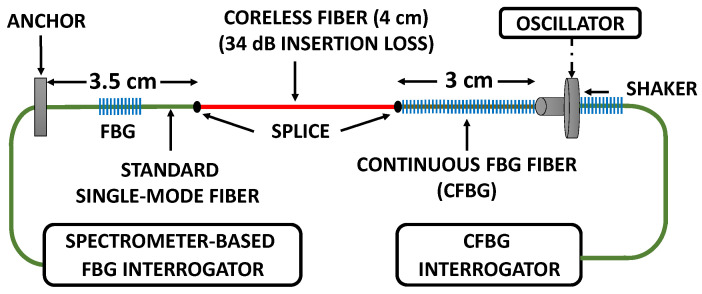
Setup for the CFBG experiment, using a polyimide-coated composite FUT. Taking advantage of the high spatial resolution of the CFBG interrogator (<1 cm), a very short CFBG fiber is used. The coreless fiber segment, serving as a high insertion loss, bidirectional isolator, allows for the CFBG interrogation to be augmented by an independent and simultaneous uniformly triggered FBG interrogation of the FUT vibrations.

The experimental setup of Figure 12 uses a FUT, comprising a polyimide-coated fused concatenation of three types of fibers: 3 cm of CFBG on its right, 3.5 cm (between the anchor point and left splice) of SMF28 with a single FBG inscribed in its middle on its left, and in-between 4 cm of a coreless fiber, providing optical isolation between the two. This FUT allows simultaneous interrogation from its two sides: from the right with the CFBG interrogator and from its left with the spectrometer-based uniformly triggered interrogator of Section 4. The coreless section, of a measured insertion loss of 34 dB, was verified to prevent mutual interference between the two interrogators. The sampling rates of both interrogators was set to be the same, 100 Hz, measurement time to 10 s and the FUT was sinusoidally vibrated under four different combinations of scaled frequencies $\xi =f_{signal}/f_{scan}\;$ (0.1 and 0.2) and scaled amplitudes $\eta =\nu_{signal-amp}/\nu_{scan}^{span}\;($0.18 and 0.38).

### 5.3. CFBG Interrogation—Results and Discussion

**Figure 13:**
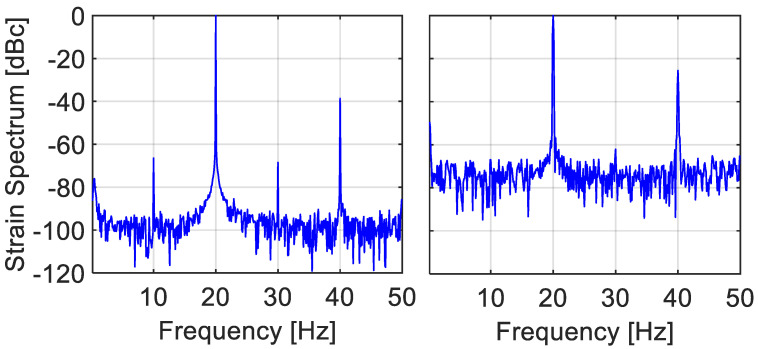
Hamming-weighed FFT spectra of the strain of the vibrating fiber in Figure 12, simultaneously measured by the two interrogators. The scaled vibration frequency and amplitude were $\xi =f_{signal}/f_{scan}=0.2$ and $\eta =\nu_{signal-amp}/\nu_{scan}^{span}=0.38$. (**Left**) Results from the uniformly triggered interrogator of Section 4. (**Right**) Results from the frequency-scanning CFG interrogator. While its noise level is higher, the peaks at 10 and 30 Hz are still barely seen (these <−60 dB peaks are not folded harmonics, but are rather due to the insufficient spectral purity of the oscillator-shaker combination).

Table 1 displays the measured levels of the normalized second harmonic, obtained by the two interrogators, for the four cases (the results of the uniformly triggered interrogator appear in parentheses). Figure 13 compares spectra of measurements by the two instruments for the high-frequency, high-amplitude case. Clearly, while the CFBG interrogator exhibits fairly low levels of harmonic distortion, they are still higher than those of the uniformly triggered interrogator, and, as expected, they grow with both ξ and η (in the absence of detailed knowledge of the inner working of the instrument, comparison with simulations could not be made).

## 6. Discussion and Conclusions

In dynamic measurand scenario, non-uniform sampling is unavoidable and inherent in periodic frequency-scanning interrogators. Static signals are uniformly sampled, since the scanning waveform always meets the signal at a constant time difference from the scan start. However, as the signal frequency and span increase (with respect to the sampling frequency and scan span, respectively), so does the non-uniformity in the sampling instants. For example, in the BOTDA experiment, the normalized spread in the sampling instants, $std\left(\tau_{n}\right)/T_{signal}$ increases from 0.01 in Figure 11 (left) to 0.03 in Figure 10 (left), in line with the increase in the second harmonic level from −26 dB to −16.3 dB. Many if not all commercial frequency scanning fiber-optic interrogators report the sampled values of the measurand on a uniform temporal grid, whereas they were actually obtained on a non-uniform one. The sampling instants are not reported, nor is information about the exact time evolution of the scan, from which the instants of sampling could be possible estimated. We have shown by simulation and experiments that this common approach leads to distorted reconstruction in both the frequency and time domains. Exposing the erroneous nature of this approach is considered by us to be the main contribution of this paper. Note also that the reconstructed signal is also temporally *shifted* from its true time dependence, Figure 5, potentially leading to synchronization issues when the same physical effect is simultaneously measured by different types of sensors.

It should be noted, though, that the widely used frequency-scanning interrogators of discrete FBGs of non-overlapping reflection spectra, are normally much less affected by this type of errors. In spite of the fact that it also involves non-uniform sampling, the common scanning span is of the order of 40–100 nanometers (nm), while the dynamic range of the tested strain/temperature rarely exceeds 10 nm (~8000 micro strain/1000 °C at 1550 nm). Hence, the filling factor $\eta =\nu_{signal-amp}/\nu_{scan}^{span}\;$ is usually smaller than 0.1. Yet, Figure 4 indicates that even for such a small value of η, the scan rate must be carefully chosen to meet a prescribed low level of harmonic distortion.

Nowadays, fiber-optic distributed sensing interrogators have become commercially available, based on either Rayleigh backscattering from standard single-mode optical fibers, or higher reflections from draw-tower ‘continuous’ FBGs. In some implementations, the faster the scan, the smaller the span. Thus, users quite often work with distortion-prone, very high filling factors, η. Section 5 has examined a commercial interrogator of ‘continuous’ FBGs, exhibiting harmonic distortion that grows with ξ and η. In principle, the analysis of this paper also applies to Rayleigh-based, frequency-scanning interrogators [21]. Practically, however, the magnitude of errors critically depends on the values of ξ and η in the relevant application.

Another worthy contribution of this work is a recommendation to the manufacturers of frequency-scanning interrogators to report the sampled values, ${\left\{s_{n}\right\}}_{n=0}^{N-1}$, together with their sampling instants, ${\left\{\tau_{n}\right\}}_{n=0}^{N-1}$. Section 4 demonstrated that obtaining that type of data from the Brillouin setup allows the use of interpolation methods to obtain a much more accurate estimation of the signal values on a uniform temporal grid. Indeed, the right panes in Figure 10 and Figure 11 exhibit a substantially lower level of harmonics in comparison with their left counterparts.

In conclusion, the reporting of non-uniformly obtained measured sampled values on a uniform temporal grid results in erroneous harmonics in the frequency domain, and in distortion in the time domain. A proposed and demonstrated mitigation approach, based on the availability of the sampling instants in conjunction with post-processing algorithms, provides a sharp increase in the fidelity of the reconstructed signal.

## Figures and Tables

**Table 1:** Normalized second harmonic levels (in units of dBc) of the longitudinally vibrating fiber of Figure 12 for two scaled vibration frequencies and two scaled amplitudes. Main values are the measurements of the CFG dynamic interrogator, while those in parentheses were obtained from the uniformly triggered interrogator of the single, in-line FBG.

$\;\xi =f_{signal}/f_{scan}$	$\eta =\nu_{signal-amp}/\nu_{scan}^{span}$	
	0.18	0.38
**0.1**	−40.5 (−42)	−30 (−40.5)
**0.2**	−38.5 (−41.5)	−25.5 (−38.5)

## Data Availability

Data will be provided under conditions, and if necessary.
